# Treatment, Therapy and Management of Metabolic Epilepsy: A Systematic Review

**DOI:** 10.3390/ijms19030871

**Published:** 2018-03-15

**Authors:** Vanessa Lin Lin Lee, Brandon Kar Meng Choo, Yin-Sir Chung, Uday P. Kundap, Yatinesh Kumari, Mohd. Farooq Shaikh

**Affiliations:** Neuropharmacology Research Laboratory, Jeffrey Cheah School of Medicine and Health Sciences, Monash University Malaysia, Bandar Sunway, 47500 Subang Jaya, Selangor, Malaysia; vanessa.lee1@monash.edu (V.L.L.L.); brandon.choo@monash.edu (B.K.M.C.); chung.yinsir@monash.edu (Y.-S.C.); uday.kundap@monash.edu (U.P.K.); yatinesh.kumari@monash.edu (Y.K.)

**Keywords:** metabolic epilepsy, metabolic disorders, antiepileptic drugs, dietary therapy, seizures, cognitive function

## Abstract

Metabolic epilepsy is a metabolic abnormality which is associated with an increased risk of epilepsy development in affected individuals. Commonly used antiepileptic drugs are typically ineffective against metabolic epilepsy as they do not address its root cause. Presently, there is no review available which summarizes all the treatment options for metabolic epilepsy. Thus, we systematically reviewed literature which reported on the treatment, therapy and management of metabolic epilepsy from four databases, namely PubMed, Springer, Scopus and ScienceDirect. After applying our inclusion and exclusion criteria as per the Preferred Reporting Items for Systematic Reviews and Meta-Analyses (PRISMA) guidelines, we reviewed a total of 43 articles. Based on the reviewed articles, we summarized the methods used for the treatment, therapy and management of metabolic epilepsy. These methods were tailored to address the root causes of the metabolic disturbances rather than targeting the epilepsy phenotype alone. Diet modification and dietary supplementation, alone or in combination with antiepileptic drugs, are used in tackling the different types of metabolic epilepsy. Identification, treatment, therapy and management of the underlying metabolic derangements can improve behavior, cognitive function and reduce seizure frequency and/or severity in patients.

## 1. Introduction

Epilepsy is a brain disease which is described as having at least two reflex or unprovoked seizures which occur separately within 24 h [1]. According to the World Health Organization, approximately 50 million people live with epilepsy around the world, with an estimated 2.4 million newly diagnosed people yearly. While treating epilepsy alone already presents enormous challenges in diagnosis and management, matters are significantly compounded in the case of metabolic epilepsy, which is classified as secondary or symptomatic epilepsy since its cause is known [2,3].

Metabolic disorders can cause seizures through one of three ways: deficiency of substrates essential for cellular metabolism or membrane function, intracellular accumulation of toxic substances and alteration of intracellular osmolality [4]. The International League Against Epilepsy (ILAE) recognizes eight types of metabolic epilepsies, namely: biotinidase and holocarboxylase synthase deficiency, cerebral folate deficiency, creatine disorders, folinic acid responsive seizures, glucose transporter type 1 (GLUT-1) deficiency, mitochondrial disorders, peroxisomal disorders and pyridoxine-dependent epilepsy (PDE) [2]. However, we also found evidence for more types of metabolic epilepsies during our literature search, such as: urea cycle disorders [5], glutaric aciduria [6], molybdenum cofactor deficiency [7], non-ketotic hyperglycaemia [8], non-ketotic hyperglycinemia [9], and succinic semialdehyde dehydrogenase deficiency [10].

As in the case of epilepsy, metabolic epilepsies can also be classified into two major types depending on their cause (inherited versus acquired), though such a classification can be described as arbitrary to an extent. This is because the cause of epilepsy is typically multifactorial and the process of identifying the cause is itself subject to the degree of investigation as well as the technology available [11]. In the context of metabolism, acquired metabolic epilepsy can be the result of a dietary deficiency, dysfunction of the organs involved in the metabolism of certain substrates, malabsorption due to reasons other than genetic defects and others [12,13]. Inherited/inborn errors of metabolism (IEM) as the name suggests, are congenital metabolic disorders which result from one or more defects in the gene/s which code for the enzymes involved in converting various substrates into products. The failure of these enzymes to perform their intended function results in the accumulation of toxic substances or those that interfere with the normal functioning of the body and thus unsurprisingly can result in neurological symptoms such as seizures [14]. IEM can be classified in several different ways, such as by their pathogenetic mechanisms (energy deficiency, toxic effect and others), age of the patient at onset, type of presenting seizures or epilepsy syndrome (infantile spasms, epilepsy with myoclonic seizures and others), as well as if a metabolic approach to treatment is possible (specialized diets, supplementation and others) [15].

While metabolic epilepsy has already been explored by previous review papers published several years prior [14,16], knowledge regarding the field of medicine typically grows by leaps and bounds and thus necessitates frequent updates to ensure that healthcare professionals are kept well informed of the latest practices regarding the treatment, therapy and management of metabolic epilepsy. In addition, there appears to be discordance regarding the number of metabolic epilepsy types recognized by the ILAE and the number found in literature. Though attempts have been made to aggregate all the different types of metabolic epilepsy in the form of literature reviews [14,16], they did not mention if their literature search was systematically performed and thus it is possible that accidental omissions could have occurred. Thus, this work aims to produce an up to date systematic review of the literature evidence reporting on the treatment, therapy and management of metabolic epilepsy.

## 2. Results and Discussion

**Figure 1:**
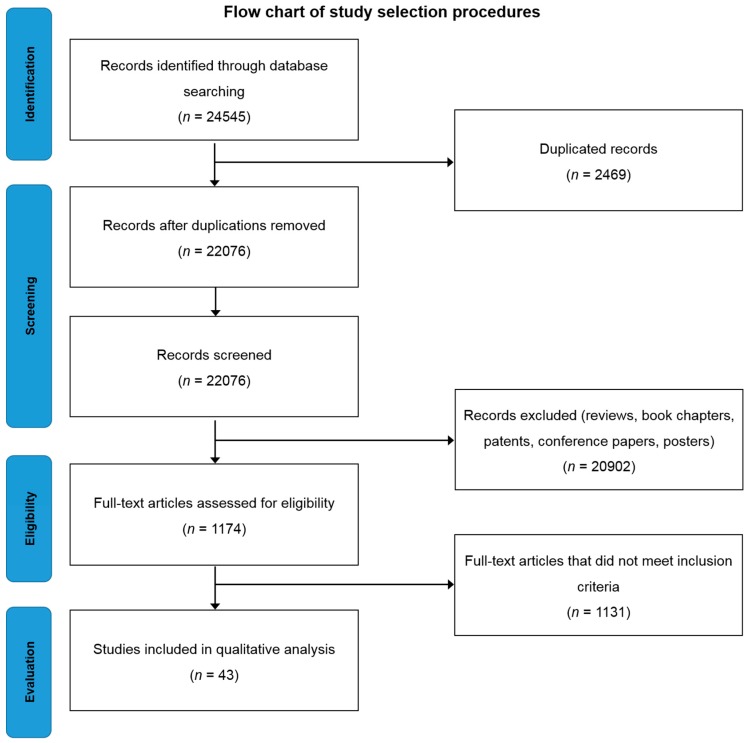
Flow chart of study selection criteria based on Preferred Reporting Items for Systematic Reviews and Meta-Analyses (PRISMA) guidelines.

Searching the selected databases with the keywords mentioned in the methodology yielded 24,545 records. After screening, the total number of articles removed was 24,502, which included; (a) 20,902 reviews, book chapters and patents, (b) 2469 duplicates, (c) 1131 which did not meet inclusion criteria (Figure 1). Forty-three eligible articles were included, compiled in Table 1 and discussed in this systematic review.

All 43 articles discussed in this review consist of either case reports or retrospective studies which only provided limited information on treatment, therapy or management of metabolic epilepsy. In addition, the numbers of articles found for each type of metabolic epilepsy were fairly limited in number overall and varied greatly from one (such as cerebral folate deficiency) to ten (glutaric aciduria) articles. There were also no randomized clinical trials, although such studies provide a stronger basis for any treatment, therapy or management strategy. The lack of randomized clinical trials and studies into the treatment, therapy or management of metabolic epilepsy in general, could stem from difficulties in conducting such trials. This is because, even if just the official ILAE list was used, there would still be eight different types of metabolic epilepsy. Thus, any study involving a single type of metabolic epilepsy would likely have to be multicentric in order to have a significant number of patients from a subset of those suffering from metabolic epilepsy, which is itself a subset of the general epileptic population (~1% of the world’s population [3]). While multicentric metabolic epilepsy studies are a worthy goal to work towards, we could not ascertain any such studies and, thus, do not have any solid data and information regarding the treatment, therapy or management of metabolic epilepsy. Hence, we have included case reports and retrospective studies despite their limitations as the alternative would be to remain oblivious. It is our hope that by analysing the studies which are currently available, several clues could emerge that may facilitate future randomized clinical trials.

### 2.1. Biotinidase Deficiency

Biotinidase deficiency is an autosomal recessive disorder which has an estimated incidence of 1:80,000 to 1:100,000 in newborns [17]. The case study by Singhi and Ray [17] is believed to be the first case of Ohtahara syndrome with biotinidase deficiency. The 3.5-month-old patient had a history of uncontrollable convulsions which were unresponsive to valproic acid (VPA) (60 g/kg/day) and phenobarbital (5 mg/kg/day). Biotinidase deficiency was only suspected when results from tests showed high anion gap metabolic acidosis with lactic acidosis, euglycemia, ketonuria and suggestive skin changes such as alopecia, despite having a normal plasma ammonia level. The patient was then treated with oral biotin (10 mg/day) which normalized his metabolic acidosis within hours and his encephalopathy and seizures disappeared within 48 h. Complete seizure control from biotin treatment for biotinidase deficiency was also found in studies by Singhi and Ray [17] and Mohamed, et al. [50]. The study by Singhi and Ray [17] showed that when biotin was discontinued for about a month, the patient developed alopecia and had low levels of serum biotinidase. Biotin was restarted and the patient was doing well and had no seizures during the follow-up at 6 years of age. He had achieved motor milestones normally, but had a speech delay. Biotin therapy is crucial in the case of biotinidase deficiency because the lack of biotinidase limits the salvage pathway for biotin recycling and this leads to a functional deficiency of carboxylase enzymes. Seizures which are accompanied by biotinidase deficiency are usually unresponsive to conventional therapies and are only responsive to pharmacological dosing of biotin, as demonstrated by the case study [17].

### 2.2. Cerebral Folate Deficiency

Cerebral folate deficiency (CFD) is a neurological syndrome associated with low levels of 5-methyltetrahydrofolate (5-MTHF), which is an active folate metabolite, in the cerebrospinal fluid, with the presence of normal folate metabolism outside the nervous system. CFD could be due to either a disturbed folate transport or an increased folate turnover within the central nervous system (CNS). CFD is an autosomal recessive disorder which manifests as severe developmental and movement disturbances, epilepsy and leukodystrophy [19]. Al-Baradie and Chaudhary [19] reported a case of two siblings from the Middle East with CFD and low cerebrospinal fluid level of 5-MTHF in the presence of normal folate metabolism outside of the CNS. As reported in the literature, both siblings were found to have a mutation in the folate receptor 1 (FOLR1) gene. This serves as an important diagnostic hallmark for CFD. Early diagnosis and management can lead to a favorable outcome. Treatment with valproate (30 mg/kg/day) and vigabatrin (50 mg/kg/day) was ineffective in both siblings. When CFD was suspected, they were treated with pyridoxine (6 mg/kg/day) and folinic acid (1.7–2.0 mg/kg/day) which led to significant improvement in development, gait, social interactions and electrocardiogram (EEG) [19].

### 2.3. Creatine Disorders

Disorders of creatine metabolism, or also known as creatine deficiency syndromes, are disorders in which the synthesis and transport of creatine (Cr) are compromised. Their hallmark is the complete absence of Cr and phosphocreatine (PCr) in the brain, which causes an array of neurological conditions such as global developmental delay, mental retardation, speech impairment, especially impaired language learning, extrapyramidal movement disorder, autism spectrum disorder and seizures [2,51].

Reports suggest that errors in Cr synthesis can lead to guanidinoacetate methyltransferase (GAMT) deficiency. This can be diagnosed by an abnormal brain magnetic resonance imaging (MRI) scan result [20]. Patients with GAMT deficiency often experience seizures which are not responsive to antiepileptic drugs [20,21]. In a case study by Bianchi, Tosetti, Fornai, Alessandri, Cipriani, De Vito and Canapicchi [20], oral Cr monohydrate treatment (400 mg/kg/day) was able to increase brain Cr levels to 40% of the normal level after 3 months and 80% at 9 months. After 16 months, brain Cr was restored to normal in gray matter and the cerebellum, but slightly less than normal in the hemispheric white matter. This also accelerated the rate of cognitive development in patient. Seizures stopped one month after oral Cr monohydrate treatment alone. Seizures did not recur, but multifocal epileptic activity was still present in the electroencephalogram [20]. Similarly, Leuzzi, Bianchi, Tosetti, Carducci, Cerquiglini, Cioni and Antonozzi [21] reported that 350 mg/kg/day of oral Cr monohydrate treatment was able to stop seizures in a GAMT deficient patient and improve overall neurological conditions [21].

### 2.4. Disorders of Urea Cycle

Urea cycle disorders (UCD) occur when a body fails to metabolize the surplus nitrogen produced by the breakdown of protein and other nitrogen containing molecules. Gupta, Kabra and Haberle [5] reported four cases of UCD in which all showed ammonia accumulation with a varying severity of enzyme defects in the urea cycle. In three out of the four cases, sodium benzoate therapy was given but this did not bring significant improvements. In addition, seizures recurred and chronic encephalopathy did not improve, which progressively led to death in all three cases. In another case, sodium benzoate therapy was also given to the patient, despite the patient not having seizures. The patient was reported to respond dramatically to the therapy, but further information was not given as the patient did not follow up [5].

### 2.5. Folinic Acid-Responsive Seizures

Folinic acid-responsive seizures are allelic to PDE and have similar biochemical markers. Research is still ongoing to investigate the mechanisms behind the responsiveness to folinic acid [2]. Lubana, Alfishawy, Singh and Atkinson [22] reported a case of seizure in a patient with elevated folate levels and suggestive findings of vitamin B12 deficiency. This is a rare case of vitamin B12 deficiency-induced generalized tonic-clonic seizures, but with elevated folate levels and no neuropsychiatric or hematologic findings except mild normocytic anemia. Since vitamin B12 is involved in myelin formation, a deficiency in vitamin B12 leads to an exaggerated effect of glutamate, which is the principal excitatory neurotransmitter, leading to epileptogenesis. The patient was given an intramuscular injection of vitamin B12 and was discharged with levetiracetam treatment, which was to be taken for 5 months. The levels of methylmalonic acid, folate and homocysteine were found to be within normal ranges. The patient remained seizure-free after stopping the intake of levetiracetam [22].

### 2.6. Glucose Transporter Type 1 Deficiency Syndrome

GLUT-1 deficiency syndrome is caused by impaired glucose transport into the brain due to a mutation in the solute carrier family 2 member 1 (*SLC2A1*) gene. It leads to neuroglycopenia which causes cognitive impairment, acquired microcephaly, epilepsy and movement disorders. Epilepsy due to GLUT-1 deficiency syndrome is usually unresponsive to standard epileptic drugs. The gold standard treatment is the ketogenic diet (KD), which promotes the use of ketones as fuel for cerebral metabolism, thereby treating the symptoms of neuroglycopenia [32,35]. KD provides brain nourishment, proper neurodevelopment, and controls seizures. However, AEDs which works via the carbonic anhydrase inhibitor mechanism, such as topiramate, zonisamide and acetazolamide, should not be used in addition to the KD because this increases the risk of acidosis and urolithiasis [32], although the use of carbonic anhydrase inhibitors was found to be effective in controlling seizures and improving EEG readings [34]. In addition, VPA was found to worsen hypocarnitinemia and inhibits fatty acid oxidation. GLUT-1 deficiency syndrome patients on KD should be given l-carnitine supplementation if hypocarnitinemia is observed [32]. A case report by Szczepanik, Terczyńska, Kruk, Lipiec, Dudko, Tryfon, Jurek and Hoffman-Zacharska [35] reported that KD effectively treats epilepsy in GLUT-1 deficiency syndrome patients as it mimics the metabolic stage of fasting, but maintains ketosis by utilizing nutritional fat rather than body fat. KD also leads to progress in myelination, which is a reflection of effective GLUT-1 deficiency management by KD [35].

Although KD has proven to be effective, it is reported that it is often challenging for families to comply with the strict diet, especially with young patients, therefore, efforts to seek alternative treatments are ongoing [32]. In 2003, Kossoff and Dorward [52] published an article about the modified Atkins diet (MAD), which was then used widely as an alternative to the conventional KD in patients of GLUT-1 deficiency syndrome to treat epilepsy. The MAD consists of approximately 10% carbohydrate, 30% protein and 60% fat without any restriction of calories or fluids. Due to the higher calorie intake, patients no longer complained of hunger [33]. The most significant benefits reported by patients were improvement in cognitive activity, reduction in seizures and ease of maintaining the diet. An increased level of vigilance, comprehension, concentration and motivation was also reported. The degree of urinary ketosis present in individuals on MAD was also less than that found in patients on conventional KD [33]. Early diagnosis is important in GLUT-1 deficiency syndrome patients to ensure that they receive proper management and treatment. The basic diagnostic hallmark for GLUT-1 deficiency syndrome is CSF hypoglycorrhachia, therefore, a lumbar puncture can be done in cases where GLUT-1 deficiency syndrome is suspected, along with a gene mutation analysis for the *SLC2A1* gene. KD or MAD should be introduced immediately once the diagnosis is made [34,35]. Mullen, Marini, Suls, Mei, Della Giustina, Buti, Arsov, Damiano, Lawrence, De Jonghe, Berkovic, Scheffer and Guerrini [36] reported a case study on three patients with myoclonic-astatic epilepsy (MAE) and *SLC2A1* mutation, suggesting that patients with MAE should be tested for GLUT-1 deficiency syndrome [36]. On the other hand, patients with paroxysmal movement disorders were reported to carry mutations in the GLUT-1 gene. They have persistent seizures which were satisfactorily controlled by valproate and carbamazepine. These AEDs, however, do not control paroxysmal movement disorders [37].

### 2.7. Glutaric Aciduria

Glutaric aciduria type 1 is an autosomal-recessive disorder resulting from a deficiency of glutaryl-CoA dehydrogenase. It is a metabolic derangement which results from the blockage of lysine, hydroxylysine, and tryptophan metabolism, leading to an accumulation of glutaric acid (GA) and increased urinary concentrations of GA and its isoforms [6,23,29]. We report one case of increased urinary GA and 3-hydroxy GA, one case of increased urinary d-2-hydroxy GA and nine cases of increased urinary l-2-hydroxy GA. We found that in most reports on glutaric aciduria, AEDs were given to control seizures. Valproate was not effective in completely controlling seizures and in some cases, only provided an initial improvement [23,24,31]. Phenobarbital and oxcarbazepine monotherapy were reported to stop seizures in patients of this category [27,28]. In most cases, polytherapy with AEDs such as levetiracetam, lamotrigine, valproate, phenytoin, oxycarbamazepine, and clonazepam, were able to completely stop the seizures [23,24,25,26]. Oral administration of carnitine and riboflavin yielded favourable results as a treatment strategy for patients with an increase excretion of GA [26,28,30,53]. Mete, Isikay, Sirikci, Ozkur and Bayram [26] also reported a case of a glutaric aciduria patient receiving oral treatment of riboflavin (200 mg/day) and l-carnitine (100 mg/kg/day) who responded with significant clinical improvement. Besides that, the use of flavin adenine dinucleotide (30 mg/day) with levocarnitine chloride was found to result in a 50% reduction in urinary l-2-hydroxyglutaric acid [30]. However, the clinical association between riboflavin and carnitine treatment and excretion of GA has yet to be sorted [53].

### 2.8. Mitochondrial Disorders

Mitochondrial disorders (MIDs) typically manifest phenotypically as epilepsy and this is also termed as mitochondrial epilepsy, according to the new classification of epilepsies by the ILAE. Finsterer and Mahjoub [2] studied 441 patients with mitochondrial disorders and found that 14% of these patients presented epilepsy and were given AEDs. Some received monotherapy, while the others received polytherapy. It was found that VPA, carbamazepine, and oxcarbazepine are toxic to mitochondria but they were still given if seizure control is acceptable and severe side effects are absent or tolerable. Because of this, other AEDs such as lamotrigine, levetiracetam, gabapentin and lacosamide are usually given to patients. Levetiracetam and lamotrigine were found to potentially exhibit protective effects against mitochondrial dysfunction. In addition to antiepileptic effects, some AEDs were found to be anti-depressive (VPA, carbamazepine, lamotrigine), anxiolytic (pregabalin) and antineuralgic (gabapentin and pregabalin). Antioxidants have also been proposed as adjunctive agents in mitochondrial epilepsy to reduce excessive oxidative stress produced by excess free radicals [2]. Myoclonic epilepsy with ragged red fibers (MERRF) and mitochondrial myopathy, encephalopathy, lactic acidosis, stroke-like episodes (MELAS) are common forms of progressive myoclonic epilepsies, which is a type of mitochondrial and metabolic disorder. In both MERRF and MELAS, patients were found to have a mutation in the transfer RNA (tRNA) gene. Zsurka, Becker, Heinen, Gdynia, Lerche, Kunz and Weber [38] reported that VPA can deteriorate the condition of patients with mitochondrial disease as it influences the mitochondrial metabolism of fatty acids and, thus, should be avoided. Levetiracetam at a dose of 2000 mg/day, clonazepam or phenobarbital monotherapy can be chosen as a treatment for mitochondrial disorders as they were found to yield seizure freedom in patients while topiramate, phenobarbital, and levetiracetam polytherapy failed to control seizures [18]. In a study by Zsurka, Becker, Heinen, Gdynia, Lerche, Kunz and Weber [38], prominent biochemical alterations and high levels of mitochondrial DNA mutations could be detected in the skeletal muscles of a patient with a CNS disorder phenotype. Therefore, histological and genetic analyses of skeletal muscle biopsies are essential for the diagnosis of myoclonic epilepsy.

### 2.9. Molybdenum Cofactor Deficiency

Molybdenum cofactor deficiency is an autosomal recessive metabolic disorder in the formation of molybdenum cofactor. Molybdenum cofactor is essential for the function of sulfite oxidase, xanthine dehydrogenase, and aldehyde oxidase enzymes. Among the methods for the definite diagnosis of molybdenum cofactor deficiency is the sulfite dipstick test and the absence of sulfite oxidase activity in skin or liver fibroblast cultures. Teksam, Yurdakok and Coskun [7] reported a female infant with molybdenum cofactor deficiency associated with Dandy–Walker malformation who presented with severe lactic acidemia and intracranial hemorrhage. The patient was given phenytoin, and then phenobarbital, pyridoxine, folinic acid, and corticosteroid were added to control seizures. Finally, the patient responded well to an intravenous infusion therapy of midazolam. However, her neurological condition deteriorated two months later, and she suffered feeding difficulties, motor and mental retardation and died of pneumonia at 4 months of age [7].

### 2.10. Non-Ketotic Hyperglycaemia

Non-ketotic hyperglycaemia is the result of elevated serum glucose levels and high plasma osmolarity, but without or with very slight ketoacidosis [54]. Pro, Randi, Pulitano, Vicenzini and Mecarelli [8] reported a non-ketotic hyperglycaemic patient presenting non-convulsive partial status epilepticus and fluctuating language disorder. In this case, EEG recording showed an episode of electroclinical seizure and continuous monitoring confirmed non-convulsive partial status epilepticus. Diazepam treatment (10 mg intravenous over 3 min) was found to reduce epileptic seizures and improve verbal communication. However, a psychiatric examination resulted in the patient being placed on carbamazepine monotherapy (600 mg/day) for its partial effects on mood stabilization. This combined treatment normalized the glucose levels and EEG readings. As a result of strict hypoglycaemic control and dietary modifications (for 10 months), the patient was seizure-free and had a normal EEG without any language disorders [8].

### 2.11. Non-Ketotic Hyperglycinemia

Non-ketotic hyperglycinemia (NKH), or glycine encephalopathy, is an autosomal recessive inborn error of glycine metabolism, which leads to severe neurological impairment and significant psychomotor disability. Glycine metabolism impairment causes the accumulation of glycine in body fluids and tissues, including those of the CNS. Thus, NKH is diagnosed by an elevated CSF: plasma glycine ratio (C: PGR) which is above 0.08 (normal < 0.04). Korman and Gutman [9] reported five cases of NKH where two patients presented with epilepsy. One of the patients had convulsions that were controlled with phenobarbitone while oral sodium benzoate and ketamine treatment improved attentiveness. However, the convulsions recurred and significant developmental delay with microcephaly, hypotonia and hyperreflexia were observed. This was due to sodium benzoate therapy that normalized plasma but not CSF glycine level, causing an increase in C: PGR. The other patient with epilepsy was initially treated with phenobarbitone, hydantoin and then clonazepam, but the treatment did not produce any significant response. Subsequent treatment with VPA and vigabatrin normalized plasma levels of electrolytes, calcium, phosphate, magnesium, lactate, pyruvate, and ammonia as well as liver and renal functions. Contradictorily, the EEG readings were highly abnormal and together with the severity of neurological impairment as well as the persistence in metabolic acidosis, they suggested the possibility of late onset NKH. The treatment was discontinued to differentiate between atypical NKH and VPA-induced elevation of CSF glycine. Upon confirmation of atypical NKH, vigabatrin treatment was reintroduced together with adrenocorticotropic hormone therapy without VPA, and this led to an improvement in seizure frequency and duration as well as EEG findings [9].

### 2.12. Peroxisomal Disorders

Peroxisomal disorders are an uncommon cause of epilepsy, usually presenting as seizures in early life, with severe neurological impairment, as defined by the ILAE. Peroxisomal disorders are a group of inherited disorders caused by mutations which lead to structural and/or functional abnormalities of peroxisomes [55]. Peroxisomal disorders can be divided into two main subgroups: (1) peroxisome biogenesis disorders [56] and (2) single enzyme defects [57]. Malformations of cortical development may co-occur with seizures in specific peroxisomal disorders, such as Zellweger syndrome and neonatal adrenoleukodystrophy. Focal seizures, generalized seizures, and epileptic spasms may occur in this condition as well [2]. There were no studies found on epilepsy due to peroxisomal disorders in our study.

### 2.13. Pyridoxine-Dependent Epilepsy/ Pyridox(am)ine-5′-phosphate oxidase Deficiency

PDE is a rare autosomal recessive disorder resulting from a deficiency of the enzyme α-aminodipic semialdehyde dehydrogenase (*ALDH7A1*), more commonly known as antiquitin (ATQ). Previously, the diagnosis of PDE was based on four criteria which were: seizures resistant to AEDs, good response to pyridoxine, complete seizure control on pyridoxine monotherapy and seizure recurrence after pyridoxine withdrawal [40,41,44]. Clinically, the diagnosis of PDE can also be done based on measurements of clinical biomarkers. Elevated urinary excretion of α-aminodipic semialdehyde (AASA) and elevated concentration of pipecolic acid in plasma and CSF are indications of PDE [41,44]. Diagnosis of PDE can also be done by mutation analysis of the *ALDH7A1* gene [39,41,42,44]. Deficiency of ATQ causes the accumulation of AASA and its cyclic form Δ1-piperidine 6-carboxylate (P6C), which forms a complex with pyridoxal phosphate (PLP), causing secondary pyridoxine deficiency and resulting in seizures [42].

The effects caused by the deficiency of pyridoxine can be reversed by the administration of pharmacological doses of pyridoxine monotherapy [18,40,44], as well as polytherapy with levetiracetam or phenobarbital [18], though the optimal dose of pyridoxine has not been established yet [40,43]. Alternatively, van Karnebeek, Hartmann, Jaggumantri, Bok, Cheng, Connolly, Coughlin, Das, Gospe and Jakobs [43] suggested the use of a lysine restricted diet as an adjunct to pyridoxine therapy. While treatment with pyridoxine compensates for PLP inactivation, it does not reduce the accumulation of substrates from lysine degradation. These potentially neurotoxic compounds could reduce the efficacy of pyridoxine and causing developmental delay or intellectual disability in 75–80% of patients. Based on a case study, a lysine restricted diet effectively reduced the chemical biomarkers of PDE and resulted in seizure control, allowing the reduction of the pyridoxine dosage [43]. A study by Alkan, Kutlu, Aslan, Sigirci, Orkan and Yakinci [46] suggested that magnetic resonance spectroscopy of the brain can be a diagnostic tool for PDE. This is because they have demonstrated the characteristic metabolic features of pyridoxine-dependent seizures to be a decreased *N*-acetylaspartate-to-creatine ratio in the frontal and parieto-occipital cortices which could represent neuronal loss [46].

Pyridox(am)ine-5′-phosphate oxidase (PNPO) deficiency is an autosomal recessive disorder of pyridoxine metabolism caused by mutations in the PNPO gene. PNPO is the rate-limiting enzyme in the synthesis of pyridoxal 5′-phosphate (PLP), the active form of vitamin B6. Patients with PNPO deficiency are resistant to AEDs and pyridoxine. In cases of PNPO deficiency, PLP should be administered promptly, after obtaining urine, plasma, and CSF samples, due to the fatality of untreated PNPO deficiency in neonates and the potential for brain damage in late onset patients. PLP successfully controlled seizures which were unresponsive to pyridoxine [39,45], but not in a case reported by Ruiz, Garcia-Villoria, Ormazabal, Zschocke, Fiol, Navarro-Sastre, Artuch, Vilaseca and Ribes [47] where PLP treatment showed an initial response for only 72 h before seizures re-appeared [47]. Excessive vanillactic acid excretion in the urine organic acid profile is the biochemical hallmark of PNPO deficiency and can be detected in first-line metabolic screening tests. Abnormalities of CSF l-3,4-dihydroxyphenylalanine (l-DOPA) and plasma amino acid levels, particularly elevated glycine or threonine, and reduced arginine, may further support the diagnosis [45].

### 2.14. Succinic Semialdehyde Dehydrogenase (SSADH) Deficiency

SSADH deficiency is an autosomal recessive disorder which results in a defect in the γ-aminobutyric acid (GABA) catabolic pathway, leading to accumulation of both GABA and 4-hydroxybutyric acid (GHB) in physiological fluids. Epilepsy was reported in approximately half of affected individuals, and thus EEG and MRI are used for diagnosis [10,48,49]. Studies showed that this enzymatic deficiency is brought about by the mutation of the *ALDH5A1* gene [10,49]. Patients with SSADH deficiency are typically treated with AEDs. Pearl, Shukla, Theodore, Jakobs and Michael Gibson [10] reported a case of a SSADH deficient patient who was initially given valproate therapy, but it caused lethargy. Then, lamotrigine was introduced and seizure control was achieved but aphasia, OCD and anxiety persisted [10]. Leuzzi, Di Sabato, Deodato, Rizzo, Boenzi, Carducci, Malaspina, Liberanome and Dionisi-Vici [48], on the other hand, reported two cases of SSADH in which vigabatrin (20 mg/kg/day) was only effective in one case [48]. An AEDs combination therapy consisting of vigabatrin, levetiracetam, oxcarbamazepine and clonazepam was reported to be effective in controlling myoclonic and tonic-clonic seizures in a patient diagnosed with SSADH deficiency [49].

## 3. Materials and Methods

### 3.1. Data Sourced and Search Strategy

A systematic search was performed using four electronic databases, namely PubMed, Springer, Scopus and ScienceDirect. The search was restricted to articles from 1 January 2000 until 30 November 2016. Search terms included “metabolic epilepsy”, “metabolic epilepsy AND therapy”, “metabolic epilepsy AND treatment” as well as “metabolic epilepsy AND management”.

### 3.2. Study Selection and Exclusion/Inclusion Criteria

The search was limited to original research articles published in English language only. Studies were selected based on the following inclusion criteria: (1) reported relevant health outcomes after successful treatment of metabolic epilepsy (metabolic epilepsy was defined as recurrent seizures due to a metabolic abnormality); (2) suggested intervention/s for effective diagnosis as part of the management. All articles with treatment or therapy attempt were included as long as the outcomes were recorded although the optimal outcome is a reduction of seizure frequency/severity or complete elimination of seizures.

We excluded duplicated articles and articles which did not meet the inclusion criteria. These included reviews, abstracts, book chapters, patents, and conference papers. They were excluded due to the lack data for assessment and comparison. Studies were excluded if there are no treatment, therapy or management reported or the outcomes were considered to be ineligible for study.

### 3.3. Data Extraction and Analysis

Three authors obtained the data independently, and then compared the titles and abstracts of each article to delete duplicated articles. Reprints of articles eligible for full-text review were obtained by reviewing the titles and abstracts after removing duplicates. After removing duplicates and articles which do not meet inclusion criteria, 1174 full-text articles were chosen and further assessed for eligibility. Only 43 articles were found to be eligible for in this study (Figure 1).

## 4. Conclusions

Based on the results of our systematic literature search, we have brought together the treatment, therapy and/or management strategies used in various case reports and retrospective studies for the treatment of 14 types of metabolic epilepsy. We believe that this work will prove valuable in updating clinicians regarding the latest developments in the treatment, therapy and/or management of metabolic epilepsy and hopefully enable them to guide metabolic epilepsy patients towards an ideal outcome. Our work could also provide useful clues to researchers to facilitate the planning of future randomized clinical trials.

## Figures and Tables

**Table 1:** Study data categorized based on the different types of metabolic epilepsy.

Type of Metabolic Epilepsy	Study Design	Intervention	Major Outcome	Reference
Biotinidase and holocarboxylase synthase deficiency	Case study (*n* = 1)	Biotin treatment (10–40 mg/day)	◦ Biotin treatment normalized metabolic acidosis within hours. ◦ Encephalopathy and seizures became passive within 48 h.	[17]
	Retrospective study (*n* = 1)	Biotin treatment	◦ Biotin treatment yielded complete control of seizures.	[18]
Cerebral folate deficiency	Case study (*n* = 2)	Pyridoxine (6 mg/kg/day) & folinic acid (1.7–2 mg/kg/day) treatment	◦ Pyridoxine and folinic acid were effective in treating intractable seizures in young children. ◦ EEG and neurological improvement was observed. ◦ Diagnosis: DNA mutation analysis- homozygous mutation in FOLR1 gene.	[19]
Creatine disorders	Case study (*n* = 2)	l-arginine (300 mg/kg/day) & Cr monohydrate (400 mg/kg/day)	◦ Blood concentrations of Cr and GAA turned out to be within normal values, thus excluding a systemic Cr synthesis deficit. ◦ Oral Cr monohydrate was given when MRI showed no increase in Cr after 2 months ◦ Brain Cr level reached 40% of the normal after 3 months and 80% at 9 months. After 16 months, brain Cr was restored to normal in gray matter and the cerebellum but was still slightly less than normal in the hemispheric white matter. ◦ Acceleration of the rate of cognitive development. ◦ Seizures stopped one month after oral Cr monohydrate treatment alone.	[20]
	Case study (*n* = 1)	Creatine monohydrate (350 mg/kg/day)	◦ AEDs were ineffective against seizures. ◦ Seizures stopped and the patient appeared less irritable and more interested in their environment after an oral dose of creatine monohydrate of 350 mg/kg/day.	[21]
Disorders of urea cycle	Case report (*n* = 4)	Sodium benzoate	◦ Patient 1 was on sodium benzoate therapy and had hyperammonemia intermittently. Patient developed intractable seizures and chronic encephalopathy which led to coma and death. ◦ Patient 2 did not have seizures although hyperammonia was present. ◦ Patient 3 presented with seizures and hyperammonemia and unfortunately died at the age of 1 week. ◦ Patient 4 had recurrent episodes of seizures and encephalopathy. Sodium benzoate was given for hyperammonemia and peritoneal dialysis was done but the encephalopathy did not improve and the patient died on day 5 of life.	[5]
Folinic acid-responsive seizures	Case study (*n* = 1)	Levetiracetam and Vitamin B12 injection	◦ Six months after beginning vitamin B12 injections, the levels of Vitamin B12, methylmalonic acid, folate and homocysteine normalized. ◦ Patient was on levetiracetam for 5 months and was off for another 5 months but remained seizure free during the period he was off Levetiracetam.	[22]
Glutaric aciduria	Case study (*n* = 1)	AEDs	◦ Valproate therapy decreased the urinary excretion of d-2-hydroxyglutarate but the excretion level was still very high. ◦ Patient died at the age of 14.5 with a history of pyloric stenosis in the neonatal period leading to surgery, epilepsy and developmental delay from the third month of life.	[6]
	Case study (*n* = 1)	AEDs Carnitine	◦ Patient was initially given carbamazepine, but was changed to valproate acid, with an apparent initial improvement. ◦ 2 months later, her seizures worsened and lamotrigine was added. ◦ She commenced regular carnitine therapy, but her seizures continued to be difficult to control and she was then commenced on levetiracetam therapy, with good clinical and electrophysiological response. ◦ She remained seizure-free for 12 months on levetiracetam and low-dose lamotrigine. ◦ A low-protein diet (restricted to 1.5 g/kg/day) was introduced. Neurological examination results remained normal, with no dystonia.	[23]
	Case study (*n* = 1)	AEDs Co-enzyme Q10 (400 mg/day) Riboflavin (200 mg/day)	◦ Partial seizure control with valproate (92µg/mL) therapy. ◦ Comedication with lamotrigine (300 mg/day) improved seizure control. ◦ Patient became seizure-free with a combination therapy of valproate, levetiracetam, and lamotrigine. ◦ Therapeutic trials with oral coenzyme Q10 (400 mg/day) for 6 months and oral riboflavin (200 mg/day) for 4 months did not produce any effects.	[24]
	Case study (*n* = 1)	AEDs	◦ Determination of the level of l-2-hydroxyglutaric acid in urine, plasma, and cerebrospinal fluid as well as a brain MRI were needed to confirm the diagnosis of l-2-hydroxylutaric aciduria. ◦ Complete seizure control was achieved following phenytoin and oxycarbamazepine therapy.	[25]
	Case study (*n* = 1)	AEDs Riboflavin l-carnitine	◦ Complete seizure control achieved from lamotrigine (5 mg/kg/day) and clonazepam (0.1 mg/kg/day) therapy but the seizures returned after a month. ◦ Complete seizure control achieved from levetiracetam (40 mg/kg/day), riboflavin (200 mg/day), and l-carnitine (100 mg/kg/day) therapy. ◦ Patient remained seizure-free for 12 months after that on lamotrigine, clonazepam and levetiracetam combination therapy.	[26]
	Case study (*n* = 1)	AEDs Folic acid	◦ Seizures stopped after treatment with phenobarbital. ◦ Folic acid supplementation (10 mg/day) started 6 months after diagnosis. ◦ However, he died 10 months later after an episode of spontaneous abdominal pain and lower gastrointestinal tract bleeding and multiorgan failure.	[27]
	Case study (*n* = 1)	AEDs Carnitine Riboflavin	◦ After diagnosis, the patient was given carnitine (100 mg/kg/day) and riboflavin (200 mg/day) which improved patient’s reflexes. ◦ When partial seizures occurred and EEG recordings showed focal epileptic discharges, oxcarbazepine (20 mg/kg/day) treatment was started and it significantly improved her seizures. ◦ She continued the oxcarbazepine treatment and remained seizure-free till date.	[28]
	Case study (*n* = 3)	AEDs	◦ One patient had complete relief from epileptic seizures with phenobarbital (50 mg/day) treatment. ◦ Another patient had partial relief from myoclonic jerks with clonazepam (0.5 mg, 3 times a day) treatment.	[29]
	Case study (*n* = 3)	Carnitine (100 mg/kg) Riboflavin (50–100 mg/day) AEDs	◦ All patients were advised moderate protein restriction and were prescribed oral carnitine (100 mg/kg), riboflavin (50–100 mg/day) supplementation, and symptomatic treatment for epilepsy. ◦ No further worsening was documented with the use of carnitine and riboflavin.	[30]
	Case study (*n* = 2)	AEDs	◦ Patient 1 suffered from recurrent seizures despite initial treatment with phenobarbital and VPA. ◦ Seizure control was achieved with a combination of VPA and primidone. ◦ Patient 2 suffered from seizures during childhood until the age of 5. ◦ Her parents stopped all treatments without any recurrence of seizures.	[31]
GLUT-1 deficiency	Retrospective study (*n* = 87)	KD AEDs l-carnitine	◦ KD together with carbonic anhydrase inhibitors increased the risk of acidosis and urolithiasis and therefore they should not be used together. ◦ Valproate worsens hypocarnitinemia, inhibits fatty acid oxidation and cannot be combined with KD. ◦ l-carnitine supplementation is given if hypocarnitinemia is observed.	[32]
	Case study (*n* = 6)	Modified Atkins diet (MAD)	◦ Reduced frequency of epileptic seizures. ◦ Improvement of cognitive activity. ◦ Urinary ketosis less than that achieved with the ketogenic diet.	[33]
	Case study (*n* = 8)	MAD AEDs	◦ MAD was found to be as effective as the ketogenic diet. ◦ AEDs with a carbonic anhydrase inhibitor mechanism (acetazolamide, zonisamide) was able to control seizures and improve EEG readings. ◦ Recommended diagnosis methods: lumbar puncture and *SLC2A1* gene analysis/sequencing.	[34]
	Case study (*n* = 3)	KD AEDs	◦ KD can effectively treat epilepsy due to GLUT-1 deficiency. ◦Basic diagnostic hallmark: CSF hypoglycorrhachia and glucose ratio below 0.6	[35]
	Case study (*n* = 3)	KD	◦ GLUT-1 deficiency should be suspected in all patients with MAE though clinical or EEG features cannot be used to exclude the diagnosis. ◦ On the other hand, the presence of PED should heighten suspicion of GLUT-1 deficiency. ◦ KD is likely to improve cognitive outcome as GLUT-1 deficiency is often associated with intellectual impairment which arises from this metabolic defect.	[36]
	Case study (*n* = 3)	AEDs	◦ Seizures persisted over time, but were satisfactorily controlled by antiepileptic drugs (valproate, carbamazepine). ◦ All patients were reported to have paroxysmal movement disorders that did not go away after AED treatment.	[37]
Mitochondrial disorders	Retrospective study (*n* = 7)	AEDs	◦ Complete seizure control with clonazepam and phenobarbital monotherapy ◦ Partial seizure control with phenobarbital + clonazepam polytherapy ◦Topiramate, phenobarbital, and levetiracetam polytherapy is ineffective.	[18]
	Retrospective study (*n* = 441)	AEDs	◦ VPA, carbamazepine, and oxcarbazepine cause mitochondrial toxicity. ◦ Levetiracetam, lamotrigine, and lacosamide are effective treatments ◦ Antioxidants are proposed as adjunctive agents to reduce the increased oxidative stress produced by excess free radicals.	[2]
	Case study (*n* = 1)	AEDs	◦ VPA deteriorated the patient’s condition because it influenced the mitochondrial metabolism of fatty acids. ◦ Levetiracetam at a dose of 2000 mg/day yielded seizure freedom. ◦ Diagnosis: histology and genetic analyses of skeletal muscle biopsy.	[38]
Molybdenum Cofactor Deficiency	Case study	AEDs Folinic acid Pyridoxine Corticosteroid	◦ Phenytoin was initiated, and then phenobarbital, pyridoxine, folinic acid and corticosteroid to control the seizure. ◦ Patient responded well to intravenous infusion therapy of midazolam. ◦ Unfortunately, patient suffered from feeding difficulties, motor, and mental retardation and died of pneumonia at 4 months of age.	[7]
Non-ketotic hyperglycaemia	Case study (*n* = 1)	AEDs	◦ Patient was placed on carbamazepine monotherapy, titrated up to (600 mg/day). ◦ His blood glucose levels returned to normal and hypoglycaemic agents combined with strict dietary control were prescribed. ◦ Ten months later, the patient was seizure-free, without any language disorders, and had a normal EEG reading. ◦ The patient was compliant to therapy and carbamazepine was reduced to the therapeutic range (6.8 ng/mL).	[8]
Non-ketotic hyperglycinemia	Case study (*n* = 5)	AEDs Benzoate Ketamine Parenteral nutrition Adrenocorticotropic hormone therapy	◦ Patient 1 had convulsions that were controlled with phenobarbitone. Treatment with oral sodium benzoate and ketamine improved attentiveness. Convulsions recurred and significant development delay with microcephaly, hypotonia and hyperreflexia were observed. ◦ Patient 2 was a false positive. ◦ Patient 3 was initially treated with phenobarbitone, hydantoin, and then clonazepam but without any significant response and subsequently with VPA and vigabatrin. Vigabatrin treatment was reintroduced together with adrenocorticotropic hormone therapy which led to an improvement in seizure frequency and duration as well as EEG findings. ◦ Patient 4 and 5 did not have epileptic seizures.	[9]
Pyridoxine dependent epilepsy (PDE)/PNPO deficiency	Case study (*n* = 1)	AEDs Pyridoxal 5′-phosphate (PLP, 30 mg/kg/day) Cefotaxime Acyclovir	◦ AEDs such as phenytoin, phenobarbital, levetiracetam, and VPA as well as treatment with cefotaxime and acyclovir did not stop the clinical seizure ◦ PLP successfully controlled seizures. ◦ Early and accurate diagnosis (genetic diagnosis/mutation analysis) is a crucial management step.	[39]
	Case study (*n* = 4)	Pyridoxine	◦ Pharmacological doses of pyridoxine are able to control seizures	[40]
	Retrospective study (*n* = 4)	Pyridoxine and AEDs	◦ Complete seizure control from pyridoxine monotherapy and pyridoxine and levetiracetam and phenobarbital polytherapy. ◦ Partial control from pyridoxine and valproate acid polytherapy.	[18]
	Case study (*n* = 1)	-	◦ EEG monitoring could potentially be an effective diagnostic tool. ◦ In addition to the stereotypical ictal pattern which is considered as an intermediate between myoclonia and spasms, short interictal interval was highlighted as a helpful marker for the disease.	[41]
	Preclinical study	-	◦ ATQ missense mutation screening system using a recombinant lat gene from *S. clavuligerus* can contribute to the diagnostic work-up of patients suspected of PDE.	[42]
	Observational study (*n* = 7)	Lysine-restricted diet Pyridoxine	◦ Effectively reduces the chemical biomarkers: CSF AASA and pipecolic acid. ◦ Seizure were controlled, leading to a pyridoxine dosage reduction.	[43]
	Case study	AEDs Pyridoxine Pyridoxine phosphate	◦ Biomarkers: elevated urinary excretion of AASA, elevated concentration of pipecolic acid in plasma, and mutation of the *ALDH7A1* gene. ◦ Diagnosis: Thorough metabolic examination, early performance of pyridoxine trial and a pyridoxine phosphate trial.	[44]
	Case study (*n* = 1)	PLP	◦ Seizures decreased significantly in patients administered PLP, following ineffective treatment with pyridoxine. ◦ PNPO deficiency is a potentially treatable disease that may be under or misdiagnosed.	[45]
	Case study (*n* = 1)	-	◦ Magnetic resonance spectroscopy demonstrated a decreased *N*-acetylaspartate-to-creatine ratio in the frontal and parieto-occipital cortices ◦ Could potentially be an effective diagnostic tool to represent neuronal loss for the diagnosis of PDE	[46]
	Case study (*n* = 1)	PLP (50 mg/kg/day)	◦ Patient presented with neonatal epileptic encephalopathy with severe seizures which do not respond to anticonvulsant drugs or pyridoxine. ◦ Patient showed a dramatic response to PLP treatment (50 mg/kg/day). ◦ 48 h after treatment, an EEG showed the disappearance of burst-suppression patterns which were present before treatment; despite persistence of multi-focal alterations. ◦ Transient clinical improvement was observed, but convulsions re-appeared after 72 h. ◦ A fungal infection could not be eliminated and multi-organ failure occurred, leading to death at 48 days of life.	[47]
Succinic semialdehyde dehydrogenase deficiency	Case study (*n* = 1)	AEDs	◦ Patient presented with severe hyperactivity and new onset generalized convulsion. ◦ VPA therapy was given but was associated with lethargy. ◦ Then, the patient was treated with lamotrigine which resulted in fair seizure control but had persistent problems with expressive aphasia, OCD and anxiety.	[10]
	Case study (*n* = 2)	AEDs	◦ In patient 1, vigabatrin (30 mg/kg/day) partially improved PED but did not influence epilepsy and language disorder. ◦ Patient 2 had sporadic generalized seizures and under vigabatrin (20 mg/kg/day), gait clumsiness and exercise-induced dystonia disappeared. ◦ Patient 2 remained seizure free for 1 year. Sertraline (1 mg/kg/day) improved OCD and after 16 months of vigabatrin treatment, his clinical condition remained stationary.	[48]
	Case report (*n* = 1)	AEDs	◦ Succinic semialdehyde dehydrogenase deficiency was diagnosed after mutation analysis of the *ALDH5A1* gene showed heterozygous mutations. ◦ Myoclonic and tonic-clonic seizures were controlled with vigabatrin, levetiracetam, oxcarbazepine and clonazepam.	[49]

EGG, electrocardiogram; FOLR1, folate receptor 1; MRI, magnetic resonance imaging; Cr, creatine; AEDs, anti-epileptic drugs; VPA, valproic acid; GLUT-1, glucose transporter type-1; KD, ketogenic diet; MAE, myoclonic- astatic epilepsy; MAD, modified Atkin’s diet; *SLC2A1*, Solute carrier family 2 member 1; CSF, cerebrospinal fluid; PED, paroxysmal exertional dyskinesia; ATQ, antiquitin; PDE, Pyridoxine dependent epilepsy; *ALDH7A1*, Aldehyde Dehydrogenase 7 Family Member A1; AASA, *α-aminodipic semialdehyde*; PLP, pyridoxal phosphate; PNPO, Pridox(am)ine-5′-phosphate oxidase; OCD, obsessive compulsive disorder; *ALDH5A1*, Aldehyde Dehydrogenase 5 Family Member A1.

## Abbreviations

5-MTHF	5-methyltetrahydrofolate
AASA	α-aminodipic semialdehyde
AEDs	Anti-epileptic drugs
*ALDH5A1*	Aldehyde Dehydrogenase 5 Family Member A1
*ALDH7A1*	Aldehyde Dehydrogenase 7 Family Member A1
ATQ	Antiquitin
Cr	Creatine
CFD	Cerebral folate deficiency
CNS	Central nervous system
C: PGR	CSF: Plasma glycine ratio
CSF	Cerebrospinal fluid
EEG	Electroencephalogram
FOLR1	Folate receptor 1
GA	Glutaric acid
GAA	Guanidoacetic acid
GAMT	Guanidinoacetate methyltransferase
GLUT-1	Glucose transporter type-1
HGA	Hydroxyglutaric aciduria
IEM	Inborn errors of metabolism
ILAE	International League Against Epilepsy
KD	Ketogenic diet
l-DOPA	l-3,4-dihydroxyphenylalanine
MAD	Modified Atkins diet
MAE	Myoclonic-astatic epilepsy
MELAS	Mitochondrial myopathy, encephalopathy, lactic acidosis, stroke-like episodes
MERRF	Myoclonic epilepsy with ragged red fibers
MIDs	Mitochondrial disorders
MRI	Magnetic resonance imaging
NKH	Non-ketotic hyperglycinemia
OCD	Obsessive compulsive disorder
PCr	Phosphocreatine
PDE	Pyridoxine-dependent epilepsy
PED	Paroxysmal exertional dyskinesia
Phe	Phenylalanine
PLP	Pyridoxal phosphate
PNPO	Pridox(am)ine-5′-phosphate oxidase
PRISMA	Preferred Reporting Items for Systematic Reviews and Meta-Analyses
*SLC2A1*	Solute carrier family 2 member 1
UCD	Urea cycle disorders
VPA	Valproic acid
